# MPIC: Molecular Prognostic Indicators in Cirrhosis Database for Clinical Context-Specific *in Silico* Prognostic Biomarker Validation

**DOI:** 10.3389/fgene.2019.00830

**Published:** 2019-09-18

**Authors:** Shun H. Yip, Naoto Fujiwara, Jason Burke, Anand Shetler, Celina Peralta, Tongqi Qian, Hiroki Hoshida, Shijia Zhu, Yujin Hoshida

**Affiliations:** ^1^Liver Tumor Translational Research Program, Simmons Comprehensive Cancer Center, Division of Digestive and Liver Diseases, Department of Internal Medicine, University of Texas Southwestern Medical Center, Dallas, TX, United States; ^2^Department of Gastroenterology, Graduate School of Medicine, The University of Tokyo, Tokyo, Japan; ^3^Broad Institute of MIT and Harvard University, Cambridge, MA, United States; ^4^Department of Genetics and Genomic Sciences, Icahn School of Medicine at Mount Sinai, New York, NY, United States

**Keywords:** prognostic prediction, study design, molecular signature, chronic disease, cirrhosis

## Abstract

Prognostic biomarkers are vital in the management of progressive chronic diseases such as liver cirrhosis, affecting 1–2% of the global population and causing over 1 million deaths every year. Despite numerous candidate biomarkers in literature, the costly and lengthy process of validation hampers their clinical translation. Existing omics databases are not suitable for *in silico* validation due to the ignorance of critical factors, i.e., study design, clinical context of biomarker application, and statistical power. To address the unmet need, we have developed the Molecular Prognostic Indicators in Cirrhosis (MPIC) database as a representative example of an omics database tailored for prognostic biomarker validation. MPIC consists of (i) a molecular and clinical database structured by defined disease context and specific clinical outcome and annotated with employed study design and anticipated statistical power by disease domain experts, (ii) a bioinformatics analysis engine for user-provided gene-signature- or gene-based prognostic prediction, and (iii) a user interface for interactive exploration of relevant clinical cohort/scenario and assessment of significance and reliability of the result for prognostic prediction. MPIC assists cost-effective prognostic biomarker development by facilitating the process of validation, and will transform the care of chronic diseases such as cirrhosis. MPIC is freely available at www.mpic-app.org. The website is implemented in Java, Apache, and MySQL with all major browsers supported.

## Introduction

Management of chronic diseases is a considerable economic burden to the medical care systems. For example, progressive fibrosis in solid organs is one of the major life-limiting chronic disease conditions associated with at least one-third of deaths worldwide (Rockey et al., 2015). Liver cirrhosis is one of the major fibrotic conditions that costs >$12 billion even in the U.S. alone (Ge and Runyon, 2016; Fujiwara et al., 2018). Organ fibrosis progression generally takes decades and the rate of disease progression is highly variable across the patients. Therefore, prognostic prediction is critical to allocate limited medical resources to rapid progressors who most need intervention, while sparing the resources for slow progressors to maximize the cost-effectiveness of patient management. However, development of prognostic biomarker is extremely challenging as evidenced by the absence of clinically translated biomarkers despite years of research (Goossens et al., 2015). This is primarily due to requirement of lengthy and costly clinical validation of candidate biomarkers, which does not fit within the budget and time frame of typical clinical trial. A fast and cheap alternative strategy of prognostic biomarker validation is sorely needed.

Publicly available omics profiles of clinical specimens may provide the opportunity of *in silico* validation for candidate prognostic biomarkers and spare resources and efforts wasted for unsuccessful clinical trials. However, currently available databases do not meet the need because the following two critical issues for prognostic biomarker assessment are disregarded (Chen et al., 2014): (1) Study-design-related information is missing. Clinical prognostic information, defined as time to clinical event, is generally incomplete due to insufficient observation period and/or biases in patient enrollment and treatment and follow-up protocols. Therefore, observed prognostic association is vulnerable to flaws in study design that could lead to false positive or negative finding (Goossens et al., 2015). Clinical patient cohort can be assembled in either retrospective or prospective manner. A retrospective cohort is a collection of patients from previously performed clinical care, where patient inclusion/exclusion criteria cannot be optimized because the enrollment is already completed in the past. In contrast, a prospective cohort is a collection of patients from future clinical care, which can be enrolled based on pre-determined inclusion/exclusion criteria, although completion of patient enrollment and follow up will take long time and is costly. In reality, virtually most of omics data suffer from the issue of biased patient enrollment because of the use of “samples of convenience,” i.e., readily available biospecimens retrospectively collected without predetermined intention of prognostic biomarker assessment (Simon et al., 2009). Thus, it is critical to annotate cohort/dataset for study design quality according to reporting guidelines to provide clue to reliability of observed prognostic association (Mcshane et al., 2006; Vandenbroucke et al., 2007); (2) Specific clinical context or scenario for biomarker application is missing. There is no clinical utility for a prognostic biomarker without specific indication of its use in real-world clinical practice, e.g., prediction of liver cancer development in Child-Pugh class A compensated viral cirrhosis patients monitored under biannual liver cancer screening, prediction of cancer-related death after 8-week cisplatin-based chemotherapy in stage III ovarian cancer.

To meet the unmet need by addressing the two major issues, we have developed Molecular Prognostic Indicators in Cirrhosis (MPIC) database as a proof of concept specifically designed for reliable prognostic assessment of candidate biomarkers using chronic fibrotic liver diseases as representative example. This scheme is readily applicable to other chronic diseases.

## Methodology and Results

Genome-wide transcriptome datasets and associated clinical outcome data are from our previous and ongoing studies as well as private contribution. Although available data are still scarce, cohorts/outcomes from public databases such as NCBI Gene Expression Omnibus (www.ncbi.nlm.nih.gov/geo) and EBI ArrayExpress (www.ebi.ac.uk/arrayexpress) are included.

The database currently contains 66 unique cohorts/outcomes of 5,540 subjects with unique clinical contexts, covering the major chronic liver diseases (i.e., viral or metabolic chronic hepatitis, cirrhosis, and cancer) for two types of outcome, time-to-event and binary outcomes (**Table 1**). The contents are curated and thoroughly annotated for study design by disease domain experts (NF and YH). The metadata include clinical demographics such as disease etiology, patient race/ethnicity, geographic region/country, median and interquartile range of clinical follow-up time, and % of patients who experienced clinical outcome of interest. Mode of patient enrollment is presented as prospective, retrospective to indicate the reliability of outcome association derived from the cohort. For instance, the analysis result from a prospective cohort can be reported as derived from “prospective-retrospective” study design, which indicates higher reliability compared to a result from retrospective study (Simon et al., 2009). Setting of patient enrollment is indicated as population-, community-, or hospital-based to explicitly indicate applicable clinical setting. Statistical power to detect certain magnitude of prognostic risk distinction is also provided to inform users about potential lack of statistical power for user-provided prognostic gene(s) at hazard ratios of 2.0 to 5.0 in Cox regression modeling, cutoffs often adopted to determine clinically meaningful prognostic risk distinction. Specific clinical contexts of biomarker application are unequivocally defined, and user can interactively find a clinical scenario of interest (see Step 1 in the next section).

**Table 1:** Clinical demographics of subjects in MPIC database.

Clinical characteristic	
Age, median (IQR)	57 (50–65)
Sex, male no. (%)	4,035 (72.8)
Race/ethnicity, no. (%)	
Asian	3,369 (60.8)
Black	31 (0.6)
Caucasian	2,078 (37.5)
Hispanic	46 (0.8)
Disease etiology, no. (%)	
Hepatitis B	1,278 (23.0)
Hepatitis C	2.699 (48.7)
Alcohol	796 (14.4)
Non-alcoholic fatty liver disease	585 (10.6)
Observation time (yr), median (IQR)	2.9 (1.8–5.2)
Observation clinical events (%), median (IQR)	40 (31–55)

MPIC consists of the following three components: (i) MySQL database of molecular profiles and clinical annotations for each specific clinical outcome in each patient cohort, (ii) bioinformatics data analysis engine developed based on GenePattern genomic analysis environment (Reich et al., 2006), and (iii) a user interface implemented using Java Grails, communicating with the database and analysis engine. Biostatistical analysis methods are implemented using the R statistical language (www.r-project.org).

**Figure 1:**
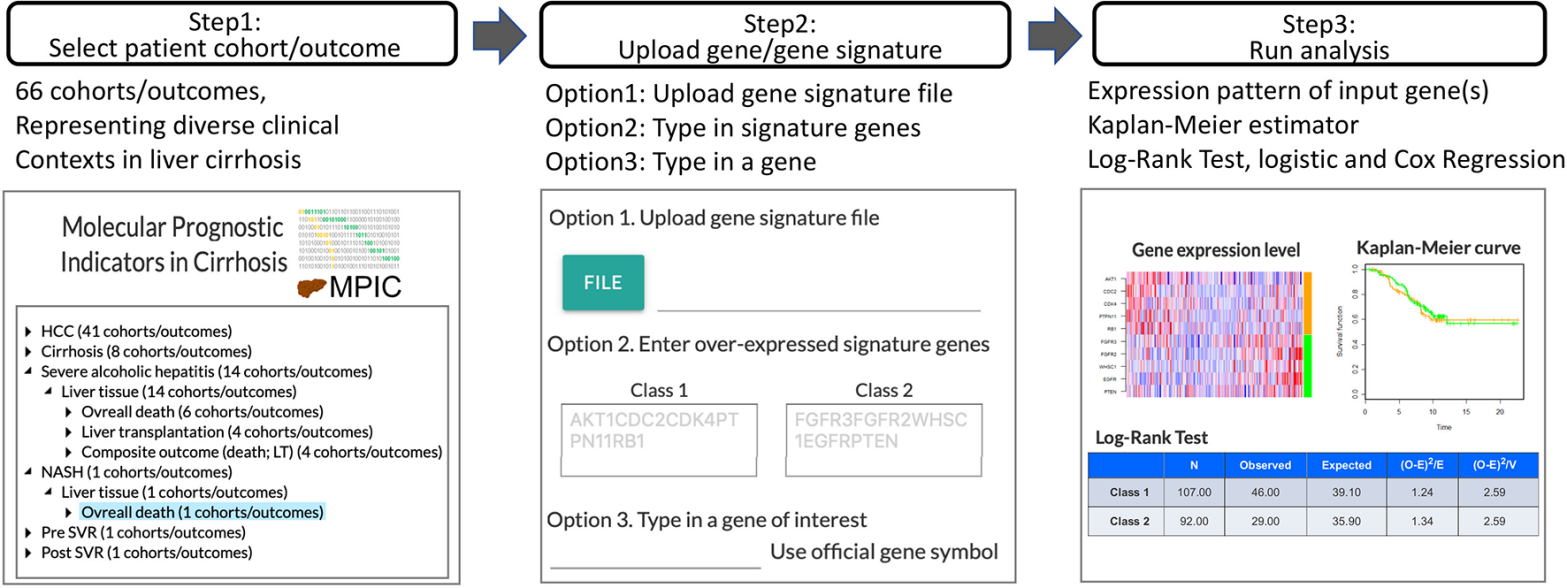
Workflow of MPIC for clinical context-specific *in silico* prognostic biomarker validation in cirrhosis.

In MIPC, users can test their own candidate prognostic gene(s) for association with a specific clinical outcome in a patient cohort following the steps described below (**Figure 1**). MPIC helps circumvent the lengthy and costly process of biomarker validation by providing opportunity to quickly perform *in silico* assessment of candidate biomarkers without requiring any clinical and experimental resources.

### Step 1: Select Patient Cohort and Clinical Outcome

Genome-wide molecular profiles of patient cohorts are hierarchically organized by disease condition (e.g., hepatocellular carcinoma, cirrhosis, alcoholic hepatitis), type of specimens (e.g., liver tissue, tumor tissue, serum), and clinical outcome (e.g., development of organ decompensation, diagnosis of stage I cancer within 2 years after surgical therapy, overall death). By selecting a patient cohort under a clinical outcome, a user can browse detailed metadata/annotations for the cohort. The cohort meta-data are summarized in **Supplementary Table 1**.

### Step 2: Upload User-Defined Prognostic Gene or Molecular Signature

Subsequently, a user-defined prognostic molecular signature or gene is uploaded. A prognostic molecular signature is defined as two sets of genes, up- or down-regulated in association with the clinical outcome of interest, in official gene symbols. Alternatively, a single gene symbol can be provided to examine association of the gene’s expression level with the clinical outcome of interest. MPIC currently supports only 2-class gene signature, i.e., two sets of genes overexpressed in association with either “Class 1” or “Class 2,” corresponding to opposite clinical outcomes such as “poor survival” or “good survival,” respectively.

### Step 3: Patient Classification and Assessment of Prognostic Association

Using the user-defined molecular signature, each patient in the selected cohort is classified into either “Class 1” or “Class 2” subgroup (e.g., “poor survival” or “good survival” subgroup) by a nearest neighbor-based versatile class prediction algorithm, Nearest Template Prediction (NTP) using cosine distance as dissimilarity metric (Hoshida, 2010). Briefly, hypothetical representative “Class 1” and “Class 2” templates are defined as vectors with the same length with the user’s input gene signature, where “Class 1” genes are set to 1 and “Class 2” genes are set to 0 for the “Class 1” template and vice versa for the “Class 2 template. Classification of each patient is performed based on proximity to either of the templates measured by cosine distance. Expression pattern of the user-provided molecular signature in the cohort is visualized as a heatmap of sample-wise Z-score for each gene. Alternatively, when a single gene symbol is provided as input, subjects are classified into high- or low-expression groups based on top quartile cut-off, and visualized as a bar graph. Association of the patient classification and time-to-event clinical outcome is evaluated by log-rank test and Cox regression and visualized as Kaplan-Meier curves. Correlation between each signature gene expression and selected time-to-event outcome is calculated as Cox score using the following equation adapted from previous study (Bair and Tibshirani, 2004):

(1) $$d=\frac{\Sigma_{k=1}^{K}\left(\Sigma_{t_{i}=z_{k}}x_{i}-d_{k}\Sigma_{i\in R_{k}}x_{i}/m_{k}\right)}{\sqrt{\Sigma_{k=1}^{K}(d_{k}/m_{k})\Sigma_{i\in R_{k}}{\left(x_{i}-\Sigma_{i\in R_{k}}x_{i}/m_{k}\right)}^{2}}}$$

where *i*, sample index; *k*, unique death time indices *z_1_*:*z_k_*; *x_i_*, transcript abundance in sample *i*, *t_i_*, observation time; *d_k_*, number of deaths at time *z_k_*; *m_k_*, number of samples in *R_k_* = *i*: *t_i_*> *z_k_*. Statistical significance of the statistic is measured as false discovery rate based on random gene resampling-based (*n* = *1,000*) nominal p-value and visualized as bar chart. Association with binary outcome is evaluated by 2 × 2 table statistics (e.g., sensitivity, specificity, positive/negative predictive values), Fisher’s exact test, and logistic regression. Data analysis engine was developed based on GenePattern (Reich et al., 2006), which can be easily extended to incorporate more analytic pipelines towards more advanced requirements.

Throughout the process, users do not have access to individual patient’s molecular and clinical data. This is a logistical advantage that lowers the bar to deposit clinical outcome data by mitigating data contributors’ concerns about sharing unpublished data, bleaching patient identity, and other regulatory issues. Besides ongoing regular expansion of cohort/dataset collection in the database, future developments will cover meta-analysis of prognostic associations derived from multiple patient cohorts for a molecular signature, multivariable analysis incorporating clinical prognostic factors, and comparison of prognostic performance across multiple molecular signatures.

## Discussion

Prognostic biomarker is the vital component in the management of patients with progressive and lethal chronic diseases. However, its development has been a daunting task due to the costly and lengthy process of clinical validation as evidenced by the scarce prognostic biomarker assays successfully translated to clinic. Currently available omics databases cannot accommodate the need because they disregard critical issues for clinical prognostic assessment such as study design, clinical context of biomarker use, setting of patient enrollment, statistical power, among many others.

To address the unmet need, we have developed a proof-of-concept database and web application, called MPIC. As opposed to biological hypothesis generation tools such as The Cancer Genome Atlas portal and associated databases, MPIC is specialized for prognostic biomarker validation using liver cirrhosis (cirrhosis) as a representative example that causes over one million deaths every year worldwide. It supports a quick go/no-go decision for prognostic biomarker candidates for further clinical development, avoids wasting cost and time for biomarker clinical trial, and enables revolutionarily more cost-effective prognostic biomarker development compared to the traditional strategy.

With this resource, we have successfully developed a prognostic assay implemented in FDA-approved clinical diagnostic platforms, supporting real-world clinical utility of our web application (initial discovery: (Hoshida et al., 2008), assay implementation and validation: (King et al., 2015; Nakagawa et al., 2016; Ono et al., 2017), incorporation in clinical trial as a companion biomarker: NCT02273362). Simulation-based analysis showed that personalized patient management with the prognostic assay is significantly cost-effective (Goossens et al., 2017), supporting that MPIC will have transformative biomedical impact on the dismal prognosis of cirrhosis patients. In the initial implementation, we primarily focused on gene expression datasets, but we will expand the database to cover other types of omics information such as non-coding RNA, epigenetic profiles, and DNA structural alterations. This scheme is readily applicable to other chronic diseases, and such an informatics resource will contribute to the substantial improvement of chronic disease management and patient prognosis.

## Data Availability

MPIC database is freely available at www.mpic-app.org. Website implemented in Java, Apache, and MySQL with all major browsers supported.

## Author Contributions

YH and SZ conducted and designed this study. SY, JB, AS, and CP implemented the database and web application. NF, HH, and TQ performed the data curation. YH and SZ wrote the manuscript. All authors reviewed and approved the paper for publication.

## Funding

This work has been supported by NIH/NIDDK (R01 DK099558, “Molecular Prognostic Indicators in Liver Cirrhosis and Cancer”), European Commission (ERC-2014-AdG-671231), Irma T. Hirschl Trust, and US Department of Defense (W81XWH-16-1-0363), Cancer Prevention and Research Institute of Texas (RR180016) to YH, and Uehara Memorial Foundation to NF.

## Conflict of Interest Statement

The authors declare that the research was conducted in the absence of any commercial or financial relationships that could be construed as a potential conflict of interest.
